# SOCS1 favors the epithelial-mesenchymal transition in melanoma, promotes tumor progression and prevents antitumor immunity by PD-L1 expression

**DOI:** 10.1038/srep40585

**Published:** 2017-01-12

**Authors:** R. Berzaghi, V. S. C. Maia, F. V. Pereira, F. M. Melo, M. S. Guedes, C. S. T. Origassa, J. B. Scutti, A. L. Matsuo, N. O. S. Câmara, E. G. Rodrigues, L. R. Travassos

**Affiliations:** 1 Experimental Oncology Unit, Department of Microbiology, Immunology and Parasitology, University of São Paulo, São Paulo, Brazil; 2 Recepta Biopharma São Paulo, Brazil; 3 Laboratory of Cancer Immunobiology, University of São Paulo, São Paulo, Brazil; 4 Immunology Department, Federal University of São Paulo, São Paulo, Brazil; 5 Immunotherapy Platform, Department of Immunology, MD Anderson Cancer Center, Houston Texas, USA; 6 Interdepartmental Group of Health Economics (Grides), Federal University of São Paulo, SP, Brazil; 7 Immunology Department, Biomedical Sciences Institute IV, University of São Paulo, São Paulo, Brazil

## Abstract

Silencing of SOCS1 protein with shRNAi lentivirus (shR-SOCS1) led to partial reversion of the tumorigenic phenotype of B16F10-Nex2 melanoma cells. SOCS1 silencing inhibited cell migration and invasion as well as *in vitro* growth by cell cycle arrest at S phase with increased cell size and nuclei. Down-regulation of SOCS1 decreased the expression of epidermal growth factor receptor, Ins-Rα, and fibroblast growth factor receptors. The present work aimed at analyzing the SOCS1 cell signaling and expression of proteins relevant to tumor development. An RNA microarray analysis of B16F10-Nex2 melanoma cells with SOCS1 silenced by shRNAi-SOCS1 was undertaken in comparison with cells transduced with the empty vector. Among 609 differentially expressed genes, c-Kit, Met and EphA3 cytokine/tyrosine-kinase (TK) receptors were down regulated. A significant decrease in the expression of TK receptors, the phosphorylation of mediators of ERK1/2 and p38 pathways and STAT3 (S727) were observed. Subcutaneous immunization with shR-SOCS1-transduced viable tumor cells rendered protection against melanoma in a syngeneic model, with decreased expression of PD-L1 and of matrix metallo-proteinases (MMPs) and CD-10 in those cells. The present work shows the role of SOCS1 in murine melanoma development and the potential of SOCS1-silenced tumor cells in raising an effective anti-melanoma immune response.

Malignant melanoma is the most aggressive skin cancer with increasing incidence in the past 30 years^1^,^2^. Melanoma cells are resistant to apoptosis and the frequently mutated B-RAF kinase protects them from *anoikis*^3^. Cytokine resistance in the melanoma metastatic form is an obstacle to immunotherapy. Moreover, melanoma can modulate the immune response by releasing immunosuppressive factors and immunoediting^4^. Nevertheless, monoclonal antibodies directed to immune checkpoints have recently been introduced with encouraging results in the treatment of metastatic melanoma^5^.

Mediators of cell signaling involved in melanoma development and progression, are Ras, B-Raf, MEK, PTEN, phosphatidylinositol-3 kinase, and Akt, constitutively activated^6^,^7^. Another signaling pathway activated by growth factor receptors involves the phosphorylation of signal transducers and activators of transcription (STAT). The duration and intensity of these signals are regulated by phosphatases and proteins that induce the activation of the JAK/STAT pathway, named suppressors of cytokine signaling (SOCS), and particularly SOCS1, a negative regulator of interleukin-6 (IL-6) and interferon (IFN-γ) signaling^8^.

SOCS1 has a central SH2 domain, an amino-terminal domain and a carboxy-terminal 40-amino-acid module that is known as the SOCS box^9^. The SOCS box interacts with elongin B and elongin C, cullin-5 and RING-box-2 (RBX2), which recruit E2 ubiquitin transferase^10^. SOCS-box-containing molecules probably function as E3 ubiquitin ligases and mediate the degradation of associated proteins through their N-terminal regions. SOCS1 localizes to the microtubule-organizing complex (MTOC) and may also regulate the localization of Jak1 to the MTOC-associated 20 S proteasome for degradation^11^.

Recent studies show that SOCS1 may affect other signaling pathways in addition to the classical JAK/STAT pathway. It can translocate to the cell nucleus and bind to NF-κB p65. Through ubiquitin ligase activity, it promotes p65 degradation^9^. Furthermore, the SH2 domain of SOCS1 can interact with the transactivation domain of p53 N-terminal region, whereas the SOCS Box mediate interaction with the DNA damage-regulated kinases ATM/ATR. SOCS1 colocalizes with ATM at DNA damage foci induced by oncogenic STAT5A^12^.

In some tumor models SOCS1 may act either as a tumor suppressor gene or an oncogene. Attenuation by SOCS1 of c-Met signaling in hepatocellular carcinoma and regulation of HGF (hepatocyte growth factor) signaling with reduced growth and migration rates can be a relevant mechanism of anti-tumor role of SOCS1 in the liver^13^. In the case of EGFR binding, SOCS proteins facilitated proteasomal degradation of the receptor^14^. As to the second interaction, SOCS1 deficiency increased IRS-2 expression and enhanced hepatic insulin sensitivity *in vivo*^15^.

Recently in our laboratory, a stable SOCS1 gene silencing using lentiviral particles and creating a variant of B16F10-Nex2 melanoma cell was obtained that was tested *in vitro* and *in vivo*^16^. SOCS1 silencing with short hairpin RNA affected tumor growth and the cell cycle, with arrest at the S phase and large-sized nuclei, reduced cell motility, and decreased melanoma cell invasion through Matrigel. A clonogenic assay showed that SOCS1 acts as a modulator of resistance to anoikis. In addition, downregulation of SOCS1 decreased the expression of epidermal growth factor receptor (mainly phospho-R), Ins-Rα, and fibroblast growth factor receptor^16^.

Based on the phenotype of SOCS1-silenced B16F10-Nex2 cells the present work focused on the expression of proteins relevant to tumor development and the signaling pathways related with SOCS1. The SOCS1-dependent expression of PD-L1 led us to examine the immunological response to SOCS1-silenced tumor cells and the cross-immune protection against B16F10-Nex2 melanoma cells.

## Results

### SOCS1 activates the transcription of genes involved in melanoma development.

**Figure 1:**
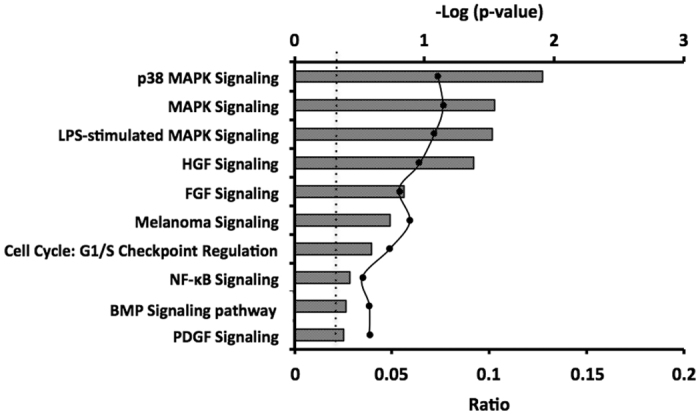
Top 10 canonical pathways related to tumor progression derived from ingenuity pathway analysis (IPA) gene ontology algorithms of down-expressed genes associated with SOCS1-silencing in B16F10-Nex2 cells. These pathways were selected following IPA “Core Analysis.” Graph shows category scores; “threshold” (dot line) indicates the minimum significance level [scored as –log (p-value) from Fisher’s exact test, set here to 0.35]. “Ratio” (differential black line and markers) refers to the number of molecules from the dataset that map to the pathway listed divided by the total number of molecules that define the canonical pathway from within the IPA knowledgebase.

Cultures permanently transfected with empty lentivirus pLKO.1 (2 chips) and with pLKO.1-SOCS1i (B16shR-SOCS1) (2 chips) were examined. SAM algorithm^17^ identified 609 genes, 178 upregulated and 431 downregulated in the cell line with reduced SOCS1 expression in comparison with pLKO.1-B16F10-Nex2 cells (Supplementary 1). Functional characterization of the differentially expressed genes using IPA (Ingenuity Pathway Analysis) showed that >60% of the genes regulated by SOCS1 protein in B16F10-Nex2 cells are involved in cell proliferation, cell cycle regulation and expression of growth factor receptors (Fig. 1). Among these genes, we selected those related to tumor progression and those displaying extreme differences in the down-regulated groups.

### SOCS1-silencing reduces the expression of tyrosine-kinase receptors

**Figure 2:**
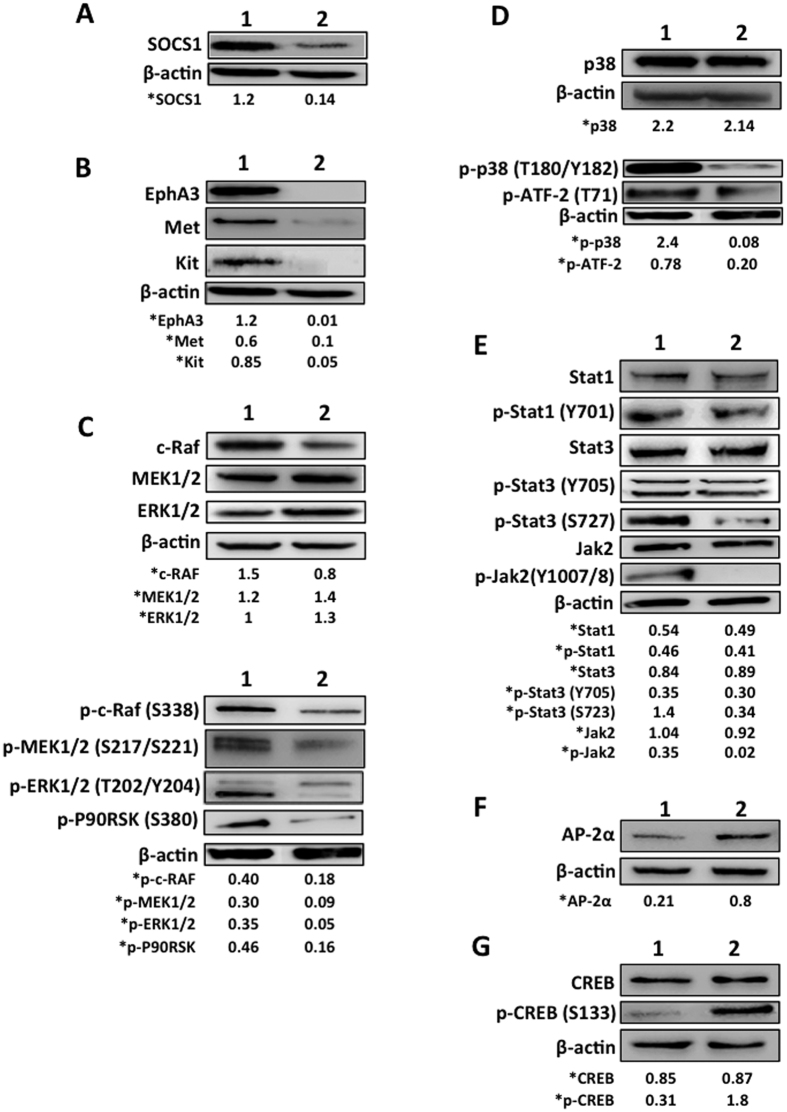
Protein expression in B16F10-Nex2 and B16shR-SOCS1 cells. Western blotting of whole cell lysates (30 μg of protein) from B16F10-Nex2 control (treated with empty lentivirus vector) (Lane 1) and B16shR-SOCS1 silenced strain (lane 2); The same total cell lysates from B16F10-Nex2 and B16sh-RSOCS1 were used for all Western blotting analysis. **(A)** Reduced expression of SOCS1 in B16F10-Nex2 cell line stably transduced with shRNAi compared to the control cell lines. SOCS1-silencing down-regulates: **(B)** tyrosine-kinase receptors; **(C)** ERK1/2 pathway, **(D)** p38 pathway and **(E)** p-Stat3 (S727) and p-Jak2 (Y1007/1008). SOCS-1-silencing up-regulates **(F)** AP-2 alpha and **(G)** p-CREB (S133). A representative blot from three independent experiments is shown for each panel. *Results were normalized by β-actin.

Analysis by Western blotting of total cell lysates from B16F10-Nex2 cells transduced with pLKO.1 and pLKO.1-SOCS1i (B16shR-SOCS1) showed that most of the identified proteins agreed with the GeneChip-hybridization results. We observed by microarray analysis a significant decrease in expression of genes of receptor tyrosine-kinases (RTKs), described as important for melanoma development^18^,^19^,^20^. The Western blotting showed that the silencing of SOCS1 protein (Fig. 2A) significantly down-regulated the expression of Kit, Met and EphA3 as compared to the controls (Fig. 2B), suggesting a functional linkage of SOCS1 and RTKs.

To confirm the possible relation of SOCS1 with the MAPK signaling via RTKs, we analyzed a few mediators of this pathway in SOCS1 silenced cells. The ERK1/2 pathway is known to play an important role in the cell proliferation of melanoma^7^. Western blotting showed that active ERK1/2 was downregulated in B16shR-SOCS1 cells. Figure 2C shows that silencing SOCS1 gene decreased total c-Raf and the phosphorylation of p-c-Raf (S338), p-MEK1/2 (S217/S221), p-ERK1/2 (T202/Y204) and p-P90RSK (S380).

Other important RTKs are the p38 MAPKs (p38α, p38β, p38δ and p38γ). Activation is mainly determined by cellular stress and inflammatory cytokines, although p38 MAPKs also regulate unrelated functions such as proliferation, differentiation and development^21^,^22^,^23^. Western blotting of B16shR-SOCS1 showed a significant reduction of p-p38 (T180/Y182) and p-ATF-2 (T71), as compared to the respective controls (Fig. 2D). These data corroborate SOCS1 role as a positive regulator of ERK1/2 and p38 MAPK pathways.

### SOCS1 and the JAK/STAT pathway in melanoma

Western blotting of lysates from SOCS1-silenced B16F10-Nex2 cells did not differ in the phosphorylation of p-Stat1 (Y701), p-Stat3 (Y705) and in the expression of Stat-3 and Jak2 as compared to controls (Fig. 2E). A significant decrease of p-Stat3 (S727) and p-Jak2 (Y1007/1008) in B16shR-SOCS1 cells was observed. It is possible that SOCS1 positively modulates the phosphorylation of Stat3 via p-ERK1/2 as shown by Chung *et al*.^24^. In contrast, Stat1 (Y701) is a poor substrate for ERK1/2. We observed in the previous results that p-ERK1/2 is downregulated in cells silenced for SOCS1 and there was no decrease in the phosphorylation of Stat1 (Y701). Decreased p-Jak2 correlated with SOCS1-silencing. In fact, interaction of SOCS1 and tyrosine-phosphorylated Jak2 stimulates proteasomal degradation of Jak2^25^.

### SOCS1-silenced cells up-regulate the Activator Protein-2α (AP-2α) and the cAMP-response-binding protein (CREB)

Since loss of expression of AP-2α is crucial in the development of malignant melanoma^26^,^27^ we determined whether silencing of SOCS1 could modulate the expression of AP-2α. Western blotting showed a 3-fold increase of total AP-2α in B16shR-SOCS1 cells (Fig. 2F) as compared to WT B16F10-Nex2 cells. Studies have also reported that CREB inhibits AP-2α expression to regulate the malignant phenotype^28^. In our system a 6-fold increase of p-CREB (Ser 133) was detected (Fig. 2G) in SOCS1-silenced melanoma cell line.

### Programmed death-ligand 1 (PD-L1) expression is reduced in B16shR-SOCS1 cells

**Figure 3:**
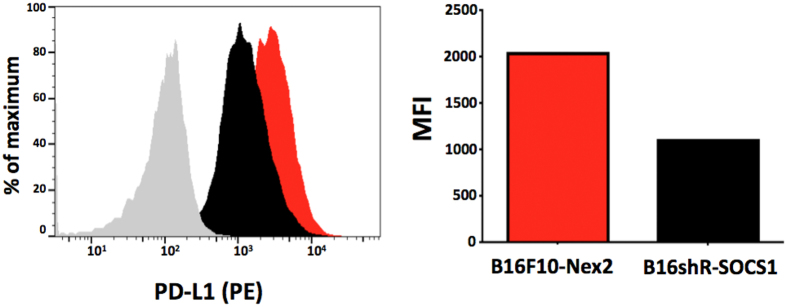
Silencing of SOCS1 reduces PD-L1 expression in B16shR-SOCS1 cells. Tumor cell lines expressing PD-L1 are shown by FACScan flow cytometer with percent values. MFI (median fluorescence intensity) values are represented on the right graph.

Previous studies have shown that the MEK/ERK signaling up-regulates PD-L1 expression and contribute to the immunosuppressive tumor microenvironment^29^,^30^. The expression of PD-L1 in SOCS1 silenced cells as examined by flow cytometry (Fig. 3) showed a significant reduction (45%) of PD-L1 expression in SOCS1 silenced cells as compared to B16F10-Nex2 cells. B16shR-SOCS1, therefore, downregulated p-ERK1/2 and the expression of PD-L1, suggesting that SOCS1 is an important immune modulator in murine melanoma.

### Prophylactic treatment with SOCS1-silenced cells

**Figure 4:**
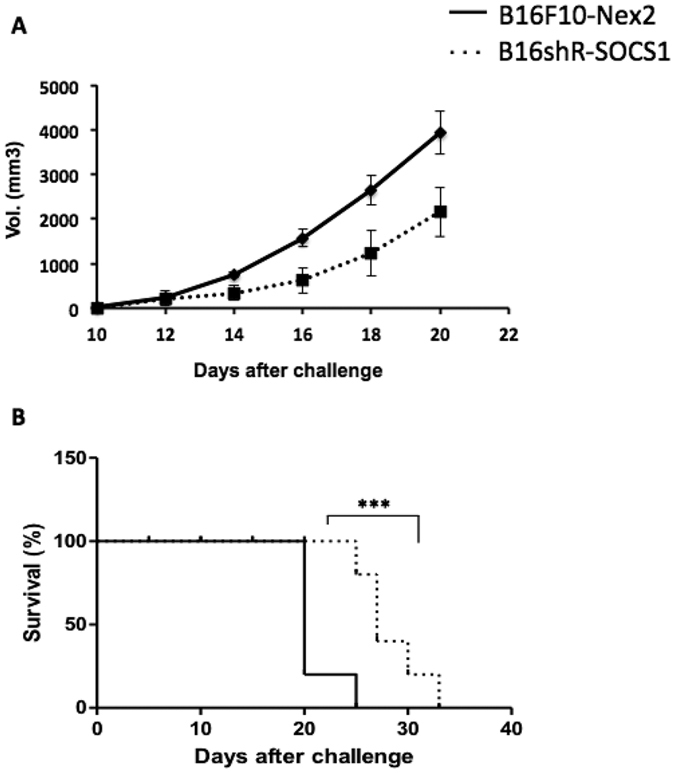
Prophylactic treatment with B16shR-SOCS1 cells. (**A**) Protection against subcutaneous B16F10-Nex2 melanoma in C57Bl/6 mice; (**B**) increase of C57Bl/6 survival. Two-way ANOVA (**** p < 0.01).

Syngeneic H-2^b^ mice were preimmunized subcutaneously in the left flanks with 5 × 10^3^ viable B16shR-SOCS1 or 5 × 10^3^ B16F10-Nex2 cells (n = 5 per group). Fifteen days after, B16F10-Nex2 viable cells (1 × 10^5^) were inoculated s.c. in the right flanks of the same animals and tumor development was monitored. Subcutaneous melanoma growth from a graft of 1 × 10^5^ B16F10-Nex-2 cells in the right flanks of mice was inhibited by 50% in animals preimmunized with B16shR-SOCS1 after 18 days (Fig. 4A). At this time period, tumor growth in untreated control animals reached the maximal allowed volume between 3,000 and 4,000 mm^3^. The survival rate of treated animals was of 80% after 25 days (Fig. 4B).

**Figure 5:**
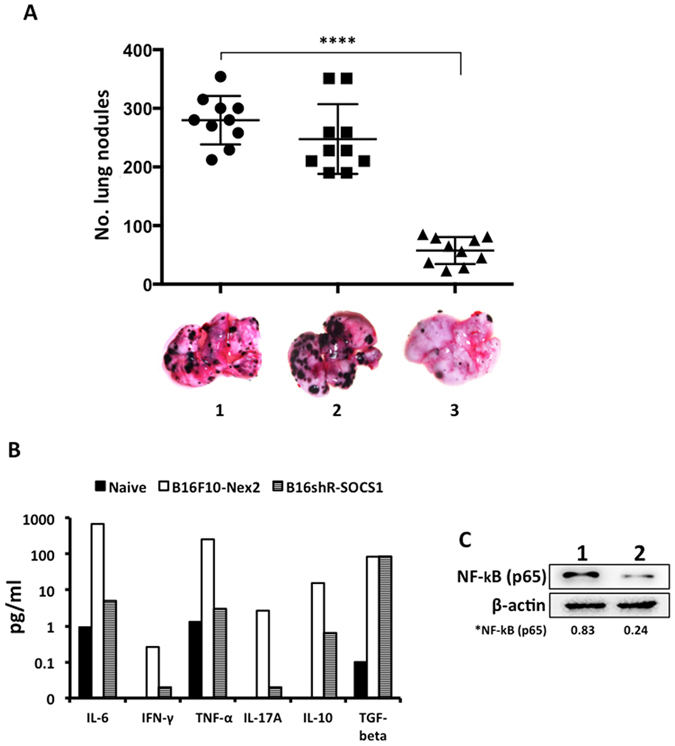
Protective effects by B16shR-SOCS1 cells, cytokine profile and NF-κB expression. (**A**) Protection against melanoma lung colonization (metastasis) in C57Bl/6 mice; 1, ●i.v. B16F10-Nex2 cells; s.c.; 2, ■B16F10-Nex2 cells, 15 days before i.v. challenge with B16F10-Nex2 cells; 3, ▲ s.c. B16shR-SOCS1 cells, 15 days before i.v. challenge with B16F10-Nex2 cells. Student-t test (****p < 0.001); **(B)** cytokine profile following B16shR-SOCS1-prophylactic immunization as shown in cultured splenocytes from mice challenged with B16F10-Nex2 cells; **(C)** SOCS1-silencing down-regulates NF-κB (p65) expression. A representative blot from three independent experiments is shown for each panel. *Results were normalized by β-actin.

The prophylactic treatment with viable B16shR-SOCS1 cells, fifteen days before challenge with WT tumor cells, was able to protect mice significantly reducing the number of lung metastatic nodules as compared to untreated animals challenged intravenously with WT B16F10-Nex2 cells (Fig. 5A). The anti-inflammatory effect of B16shR-SOCS1 prophylactic immunization was shown in cultured splenocytes from mice challenged with B16F10-Nex2 cells, with significant reduction of IL-6, TNF-α, IFN-γ, IL-17a and IL-10 (Fig. 5B). Western blotting of B16shR-SOCS1 total cell lysate showed decreased expression of the pro-inflammatory transcription factor NF-κB (p65) (Fig. 5C). These date indicate that SOCS1 plays an important role in melanoma development by immune-modulation of the tumor microenvironment.

### SOCS1-silenced subcutaneous melanoma cells induce enhanced cellular infiltrate, and elicit a CD8^+^ T cell-dependent protective immune response

**Figure 6:**
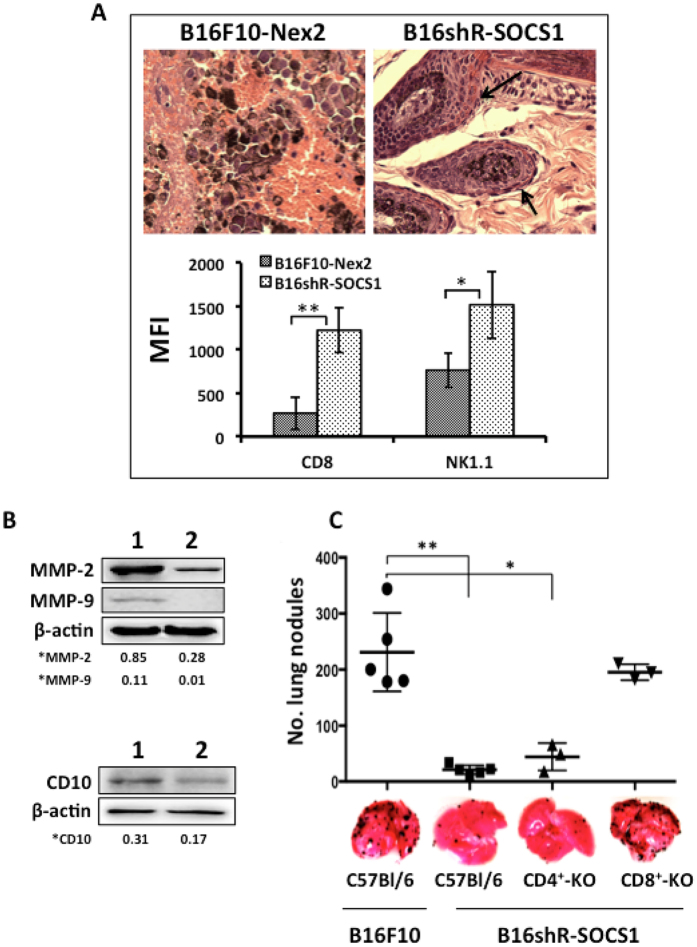
B16shR-SOCS1 cells microenvironment and protective role of CD8^+^ T lymphocytes. **(A)** SOCS1-silenced cells enhance cellular infiltrate in the s.c. tumor microenvironment (black arrows); T-CD8 and NK.1.1 cells in tumor infiltrate are shown by FACScan flow cytometer with median fluorescence intensity (MFI); (**B**) SOCS1-silencing down-regulates MMP2, MMP9 and CD10 expression; **(C) T-**CD4^+^-KO and T-CD8^+^-KO mice were submitted to prophylactic treatment with SOCS1-silenced cells and were rechallenged with WT B16F10-Nex2 cells. The treatment was not effective in T-CD8^+^-KO mice. Student-t test (*p < 0.05; **p < 0.01). A representative blot from three independent experiments is shown for each panel. *Results were normalized by β-actin.

Histology of lesions from C57Bl/6 mice injected subcutaneously with B16shR-SOCS1 cells showed enhanced cellular infiltrate, T-CD8 cells and NK1.1 cells (Fig. 6A) and reduction of tumor invasiveness. Western blotting of B16shR-SOCS1 total cell lysate showed decreased expression of MMP2, MMP9 and CD10, implicated in malignant tumor progression, as compared with B16F10-Nex2 total cell lysate (Fig. 6B). These data indicate that the expression of SOCS1 on murine melanoma cells is associated to the EMT and tumor progression phenotype.

In the metastatic model with B16F10-Nex2 cells, no significant reduction of lung nodules was observed in B16shR-SOCS1 cells injected in CD8 T-cell KO mice as compared to equally challenged C57Bl/6 WT mice. Subcutaneous injection of B16shR-SOCS1 cells in CD4 T-cell KO and WT mice promoted otherwise, a significant reduction of lung metastatic nodules (Fig. 6C), suggesting that SOCS1-silencing elicits a protective immune response against metastatic melanoma mediated by CD8^+^ T-cells.

**Figure 7:**
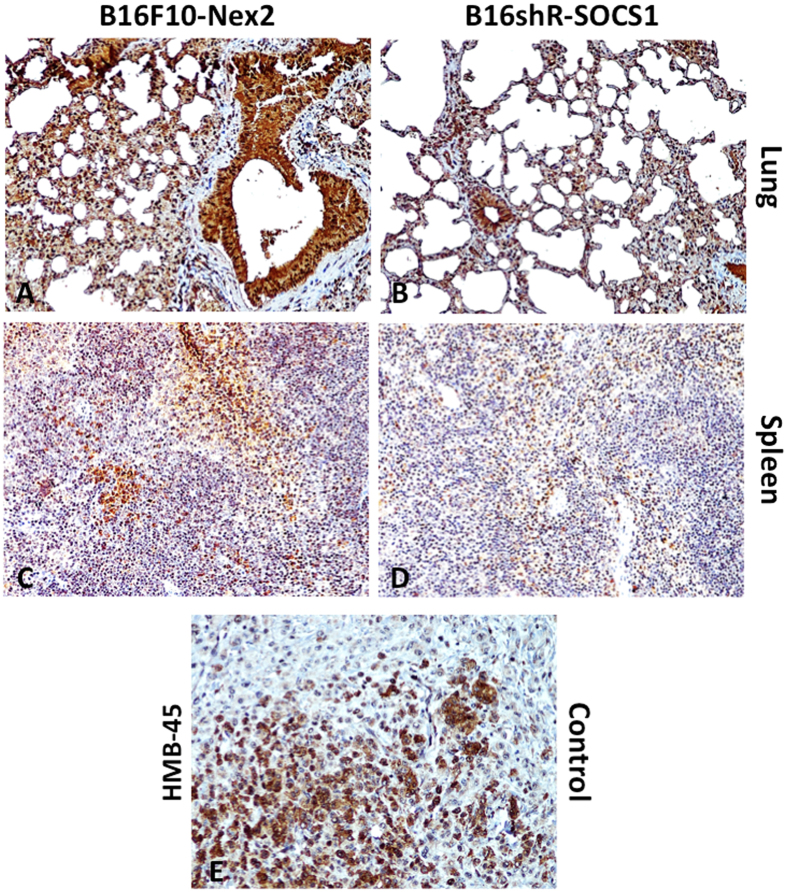
Expression of HMB-45 melanoma antigen on spleen and lung tissues from mice submitted to prophylactic treatment with SOCS1-pos and -neg cells. Immunohistochemistry of (**A**) spleen tissue from mice submitted to B16F10-Nex2 or (**B**) B16shR-SOCS1 prophylactic treatments; and (**C**) lung tissue from mice submitted to B16F10-Nex2 or (**D**) B16shR-SOCS1 prophylactic treatments; (**E**) human melanoma tissue lesions (positive control). In brown: HMB-45 staining. Counterstaining: hematoxylin; magnification,  × 200.

**Figure 8:**
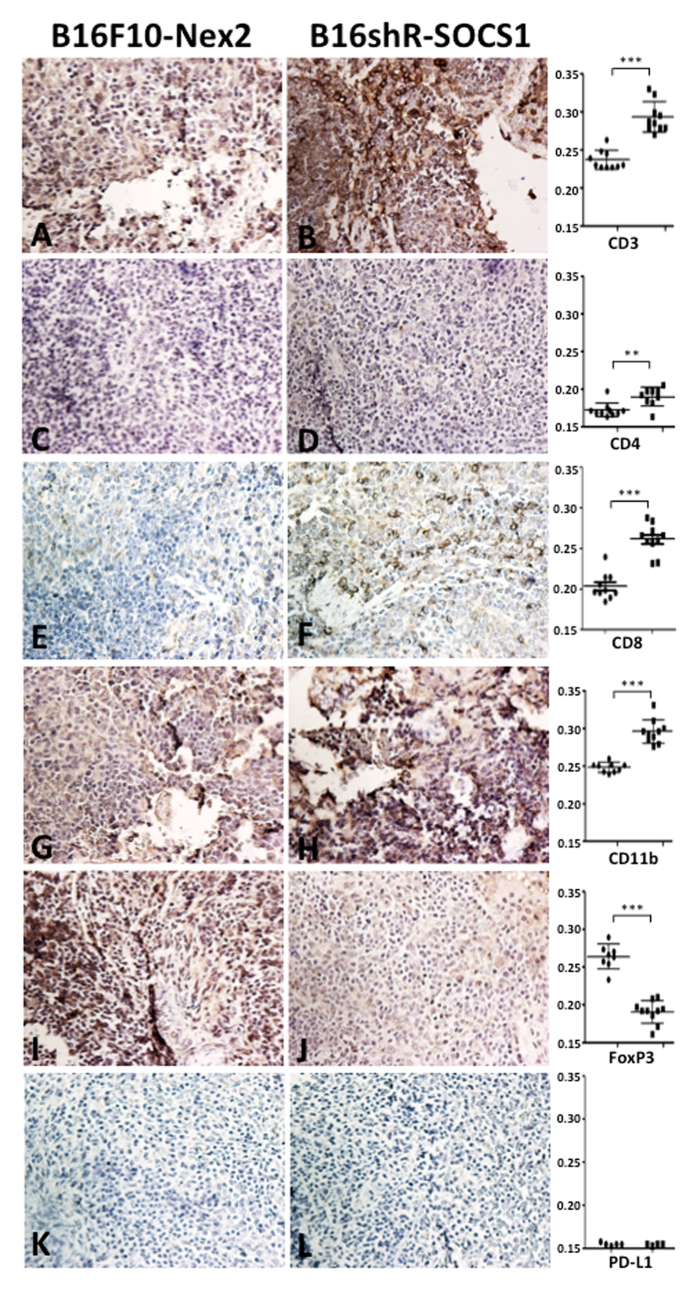
Immunohistochemistry of (I) spleen tissues from B16shR-SOCS1 prophylactic treatment. Left panels, animal tissues challenged s.c. with murine melanoma cells B16F10-Nex2 and intravenously rechallenged with the same WT tumor cells; right panels, the same protocol with primary s.c. challenge with B16shR-SOCS1 cells. (**A**,**B**) CD3 staining; (**C**,**D**) CD4 staining; (**E**,**F**) CD8 staining (**G**,**H**) CD11b staining; (**I**,**J**) CD4^+^FoxP3^+^ T cell staining and (**K**,**L**) PD-L1 staining. Counterstaining: hematoxylin; magnification,  × 200. Graphs represent optical densities of 3,3′-Diaminobenzidine (DAB) staining of 10 different fields from 3 different mice. Student t-test (**p = 0.027; ***p < 0.001).

**Figure 9:**
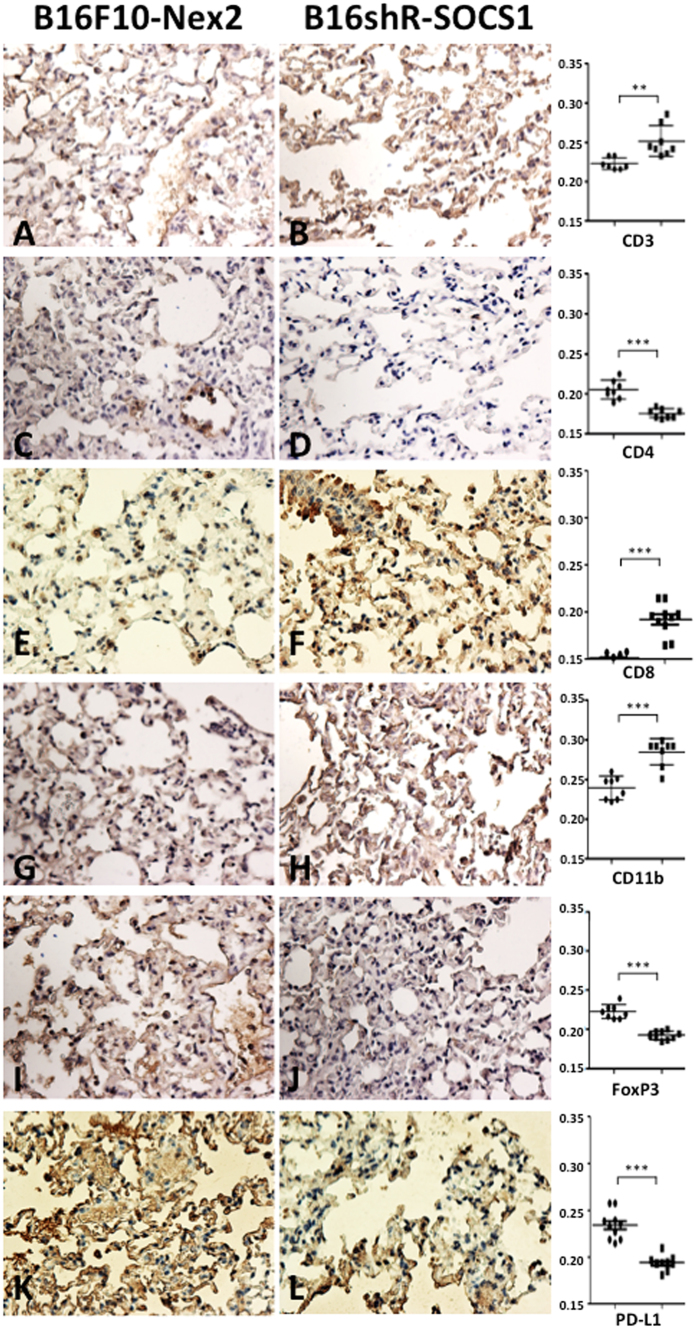
Immunohistochemistry of (II) lung tissues, from B16shR-SOCS1 prophylactic treatment. Left panels, animal tissues challenged s.c. with murine melanoma cells B16F10-Nex2 and intravenously rechallenged with the same WT tumor cells; right panels, the same protocol with primary s.c. challenge with B16shR-SOCS1 cells. (**A**,**B**) CD3 staining; (**C**,**D**) CD4 staining; (**E**,**F**) CD8 staining (**G**,**H**) CD11b staining; (**I**, **J**) CD4^+^FoxP3^+^ T cell staining and (**K**, **L**) PD-L1 staining. Counterstaining: hematoxylin; magnification,  × 200. Graphs represent optical densities of 3,3′-Diaminobenzidine (DAB) staining of 10 different fields from 3 different mice. Student t-test (**p = 0.027; ***p < 0.001).

The effects of s.c. prophylactic treatment with B16shR-SOCS1 cells, of mice submitted to intravenous rechallenge with B16F10-Nex2 cells, were examined by immunohistochemistry of lung and spleen samples. Melanoma metastatic nodules were stained by anti-HMB-45 antibody (Fig. 7). A significant reduction of Treg FoxP3^**+**^lymphocytes and increased infiltrate of CD11b, CD3 and CD8 expressing cells were observed in mice treated with B16shR-SOCS1 cells, as compared with tissues from mice submitted to prophylactic treatment of B16F10-Nex2 cells. The T-CD4^**+**^ expression was similar in both groups (Figs 8 and 9). PD-L1 expressing cells, in the SOCS1-neg prophylactic cell treatment, were significantly reduced in lung tissue colonized with tumor cells (Fig. 9). These results suggest that SOCS1 negatively regulates the antitumor effector response, mainly by inducing Treg cells through the expression of PD-L1^31^.

### SOCS1 promotes EMT through a non-SMAD dependent pathway.

**Figure 10:**
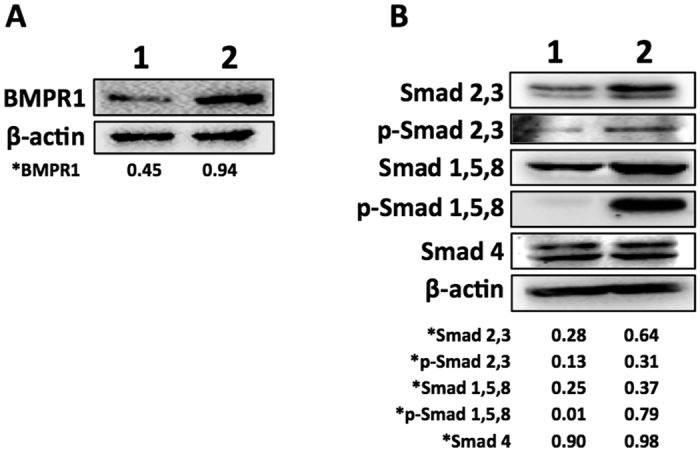
SOCS1 regulates the expression of BMPR1 and TGF-beta/BMP signaling pathways. Western blottings of whole cell lysates (30 μg of protein) from B16F10-Nex2 control cells (treated with empty lentivirus vector) (lane 1) and B16shR-SOCS1 silenced strain (lane 2). (**A**) SOCS1-silencing enhanced BMPR1 expression, and (**B**) enhanced total Smad 2/3, p-Smad 2/3, total Smad 1/5/8 and p-Smad 1/5/8. Silencing of SOCS1 did not alter the expression of Smad 4. A representative blot from three independent experiments is shown for each panel. *Results were normalized by β-actin.

Since we have shown that RTKs and ERK1/2 are upregulated by SOCS1 in murine melanoma cells and that the expression of matrix metallo-proteases (MMPs) and CD10, which are involved in the neoplastic transformation and tumor progression^32^, can occur by activation of MAPKs through a Smad independent pathway^33^,^34^, we analyzed the expression of total and phosphorylated Smad 2/3, activated by TGF-β; as well as Smad1/5/8, by BMP signaling. Western blotting showed enhanced expression of BMPR1 receptor (Fig. 10A), total and phosphorylated Smads 2/3 and Smads 1/5/8 in B16shR-SOCS1 cells, and no difference in the expression of Smad 4 as compared to controls (Fig. 10B). Results showed that SOCS1 downregulates TGF-β and BMP canonic pathways whereas activates MMP transcription factors through Smad independent activation, thus favoring EMT in melanoma cells.

## Discussion

In the present study, the expression of genes downregulated in SOCS1-silenced B16F10-Nex2 murine melanoma cells helped to characterize both the involvement of SOCS1 in the malignant context of murine melanoma and the intrinsic role of signaling pathways on the promotion of tumor progression.

The transcripts microarray of B16shR-SOCS1 identified a total of 609 differentially expressed genes (DEGS), 178 with increased expression and 431 with decreased expression. The Ingenuity Pathway Analysis (IPA) from DEGS created 10 hypothetical canonical pathways based on the down-regulated gene groups. Among these pathways, the p38 MAPK and other pathways activated by growth factors were affected by SOCS1 inhibition. Scutti *et al*.^16^ found a significant correlation between SOCS1 expression in B16F10-Nex2 melanoma cells and their aggressive tumorigenicity. Downregulation of SOCS1 in B16F10-Nex2 murine melanoma cells decreased the expression of several growth factor receptors e.g., insulin receptor α-chain, EGFR (phosphorylated), and FGFR-4 and -5 and a possible attenuation of MAPK signaling pathways would be compatible with alterations in the cell cycle S phase, decreased proliferation, and decreased resistance to anoikis, present in these cells^16^. We analyzed the expression of three receptor tyrosine-kinases (RTKs) that are involved in tumor cell growth and are overexpressed in murine melanoma cells, e.g. Kit, Met and EphA3. Western blotting showed a significant decrease in the expression of these receptors in B16shR-SOCS1 cells, which confirms the microarray data.

A major MAPK pathway related with murine melanoma is the ERK1/2 pathway associated with activation of the Ras/Raf/MEK/MAPK cascade, in which, mutations in oncogenes such as BRAF, culminate in exacerbated cell proliferation^35^. We have shown that SOCS1 upregulates ERK1/2 phosphorylation cascade and this activation is independent of p-RSK2 (Tyr 529) (Data not shown).

In cancer, ERK activation is associated to cellular proliferation, while p38 activation is related to decreased cell proliferation or pro-apoptosis phenotype. In melanoma, it has been described that both pathways are simultaneously activated with a positive feedback between ERK and p38 activation, stimulating the migration and proliferation *in vivo*^36^. Scutti *et al*.^16^ confirmed the low invasiveness of B16shR-SOCS1 in syngeneic animals. These data could possibly indicate downregulation of p38 in SOCS1-silenced cells. Indeed, p-p38 (Thr180/Tyr182) was less expressed, suggesting a SOCS1-related upstream activation of ERK1/2 and p38 MAPK pathways.

The family of ERK/MAPK proteins specifically phosphorylate STAT3 on Ser 727 in response to a regulatory hormone e.g. hepcidin in COS cells^24^. Based on these results, we analyzed phosphorylation of STAT3 (S727) in SOCS1-silenced cells and observed a significant reduction of p-STAT3 (S727). The phosphorylation of p-Stat1 (Y701) was not affected, as STAT1 is not a good substrate for ERK. Also unaffected was the phosphorylation of STAT3 at Y705.

The expression of CREB, a transcription factor, was also analyzed. Inhibition of PKA-dependent CREB phosphorylation, as well as silencing of CREB expression by shRNA, restored AP-2α protein expression in metastatic melanoma. A dual mechanism is involved in the loss of AP-2α expression: binding of CREB to the AP-2α promoter and CREB-induced overexpression of another oncogenic transcription factor, E2F-1^28^. AP-2 alpha regulates genes associated with metastasis, including growth factor receptors such as Kit. Presently, we show that Kit is poorly expressed in the SOCS1-silenced melanoma cell line. We also observed the increase of p-CREB (S133) in B16shR-SOCS1 cells as related to the low expression of SOCS1. We hypothesize that SOCS1 regulates AP-2 alpha and p-CREB (S133) expression by modulating RTKs. Recent studies demonstrated that phosphorylated CREB directly inhibited NF-κB limiting a proinflammatory response^37^.

Tumor cells have different mechanisms to escape immunological attacks. Oncogenic driver genes can create an immunosuppressive environment by secretion of inflammatory mediators including chemokines, leading to the infiltration of pro-tumor leucocytes and, as deeply investigated recently, upregulation of immune checkpoint molecules^38^. Here, we found that SOCS1 silencing therapies may subsequently influence both the immunogenicity of the tumor as well the ability of the host to mount an effective immune response. Programmed death-1-ligand (PD-L1), which negatively regulates T-cell immune responses, can be regulated by multiple signaling pathways including MAPK^39^. Studies showed that melanoma cells have increased expression of PD-L1 and that this increase is related to melanoma progression and downregulation of the immune system^40^,^41^. Jiang *et al*.^42^ reported that enhanced phosphorylation of STAT3 at Ser 727 and Tyr 705, and of c-Jun are directly involved in PD-L1 expression in human melanoma cells. Knockout of STAT3 alone or c-Jun alone is sufficient to reduce PD-L1 expression. In agreement with this observation, in the present study we found that depletion of SOCS1 in B16F10-Nex2 murine melanoma cells by using shRNAi affected PD-L1 expression. The inhibition of MAPK-ERK1/2 and decreased p-STAT3 (S727) in these cells can be associated to PD-L1 suppression. It suggests that SOCS1 inhibition may not only have effects on tumor cell properties, but also strongly regulates PD-L1 expression that could influence the tumor-induced immune response.

Immune evasion mechanisms include the interaction of PD-1 receptor with PD-L1/B7-H1 ligand^43^ and the suppression of T-CD8^+^ cells by T-CD4^+^CD25^+^FoxP3^+^ cells (Treg)^44^. Studies demonstrated that MAPKs play an important role in the development of Treg cells^45^,^46^. Francisco *et al*.^30^ showed that the expression of PD-L1 attenuates the phosphorylation of p42/ERK2, suggesting that PD-L1 may mediate the induction of Treg cells by modulating ERK2 activity and MAP kinase signaling cascade. The increase in PD-L1 expression in tumor cells can induce Foxp3^+^ Treg cells increasing the suppression of antitumor T-cell responses and thus allowing tumor progression^47^. Our data showed a systemic reduction of IL-17A and IL-10 cytokines and a significant decrease of FoxP3^+^ Treg cells in spleen and lung tissues of mice that received a subcutaneous inoculation of B16shR-SOCS1 cells.

The contribution of SOCS1 signaling to melanoma immune evasion was shown by the prophylactic s.c. inoculation of a low number of B16shR-SOCS1 viable cells, that was able to protect against rechallenge with B16F10-Nex2 melanoma cells in a syngeneic model. Tumor volume was significantly reduced and animal survival increased. The control group received a prophylactic treatment of live B16F10-Nex2 cells. Seemingly, the subcutaneous inoculation with B16shR-SOCS1, rather than B16F10-Nex2 cells, induced an effective immune response against the subsequent challenge by aggressive melanoma cells with increased T-CD8 and NK1.1. cell infiltration into the tumor stroma. Although immunogenic, SOCS1-silenced cells are unable to develop s.c. tumors as already seen by Scutti *et al*.^16^ who demonstrated the importance of SOCS1 in melanoma tumorigenicity.

Prophylactic treatment with B16shR-SOCS1-silenced cells was also effective in a metastatic melanoma model. A significant decrease in the number of nodules in the lungs of mice treated with B16shR-SOCS1 cells and challenged with B16F10-Nex2 cells was observed, as compared to the untreated control group. These results indicate that a protective immune response induced by the s.c. inoculation of B16shR-SOCS1, before challenge with WT melanoma cells, was also effective against systemic tumor development. A need for T-CD8^+^ effector cells to induce protection was shown, as inferred from the lack of it in T-CD8^+^-knockout animals. WT C57Bl/6 and T-CD4^+^-KO mice also had a significant reduction in the number of pulmonary nodules when treated with B16shR-SOCS1. A reduction of systemic pro-inflammatory cytokines, mainly TNF and IL-6, in mice treated with B16shR-SOCS1 and down-regulation of NF-κB (p65) in these cells suggest that SOCS1 could favor the epithelial-mesenchymal transition (EMT) leading to tumor progression^48^,^49^. Also, we demonstrated an increase of T-CD8^+^ tumor infiltrated lymphocytes and a decrease of PD-L1 expressing cells in B16 tumors in the lungs of mice previously inoculated with shR-SOCS1 cells. These results suggest that SOCS1 favors the tumor immune scape by PD-L1 expression on melanoma cells.

In late stages of tumorigenesis, TGF-β and BMP signaling promote tumor growth by inducing EMT through Smad-dependent effects (canonical pathway)^50^,^51^. On the other hand, EMT can be induced by Smad-independent activation through RTKs and ERK activation (non-canonical pathway)^33^,^34^. It has been described that ERK negatively regulates Smads activity through phosphorylation of Smad 2 and Smad 3 and that this inhibition favor the non-Smad pathways in TGF-β signaling^52^,^53^. Also, ERK substrates can interact and function simultaneously with Smads to regulate gene expression^34^,^53^,^54^. Our results showed that SOCS1, in B16F10-Nex2 cells, down-regulates the expression of MMPs, mainly MMP2 and 9, through activation of ERK1/2 by RTKs. Silencing of SOCS1 in murine melanoma cells resulted in Smads 2/3 and Smads 1/5/8 activation, simultaneously with the increased expression of MMPs in B16F10-Nex2 murine melanoma cells, by a non-canonical pathway.

The present data suggest that SOCS1 plays an important role on melanoma progression by favoring epithelial to mesenchymal transition, upregulating tyrosine-kinase receptors and matrix metallo-proteinases. Conversely, SOCS1-silencing favors an anti-melanoma immune response by activation of effector T-CD8+ cells against the tumor, concomitant with regulation of ERK1/2 and p38 MAPK pathways and decreased expression of PD-L1 in murine melanoma B16F10-Nex2 cells. In conclusion, shR-SOCS1 murine melanoma cells represent important immunogenic agents effective against metastatic and subcutaneous melanoma. On the other hand, our data could be useful to understand the mechanisms of tumor resistance to immune modulation therapies. Previous studies demonstrated that aberrant expression of SOCS1 in human melanoma cells directly promote cell proliferation and can be associated with poor prognosis in patients with melanoma^55^. SOCS1 expression may downregulate biological responses by endogenous and/or therapeutically administered cytokines^56^,^57^. The association of SOCS1 expression with PD-L1 further refine the selection of predictive biomarkers to anti-PD-1 based therapy, or therapies that induce antitumor immunity, in patients with metastatic melanoma. Recently, studies demonstrated that tumors from metastatic melanoma patients, who did not respond to anti-CTLA4 and anti-PD-1 therapy, had a high frequency of genomic alterations of IFN-γ pathway genes such as *IFNGR1, IFNGR2, IRF1, JAK2* coincident with amplification of IFN-γ signaling pathway suppressor genes, *SOCS1* and *PIAS4*^58^,^59^. These findings indicate that loss of IFN-γ signaling in tumor cells and SOCS1 amplification represent an important mechanism of tumor resistance to immune checkpoint therapy.

## Material and methods

### Ethics Statement

The Ethical committee of UNIFESP approved all experiments with mice in accordance with the relevant international guidelines (Fapesp Project 2010/51423-0, CEP no. 1234/11).

### Tumor cell lines and cell culture conditions

The following cell lines were used in the present study: the murine melanoma cell line B16F10-Nex2 characterized by adherence, darkly melanotic cells, fast growing *in vitro* and *in vivo*, forming black tumor masses and black nodules in the lungs when injected subcutaneously and intravenously, respectively, in syngeneic H-2^b^ C57Bl/6 mice. The original B16F10 lineage was obtained from the Ludwig Institute for Cancer Research, São Paulo branch. The B16F10-Nex2 subline was isolated at the Experimental Oncology Unit, Federal University of São Paulo (UNIFESP), and deposited at the Banco de Células do Rio de Janeiro (BCRJ), reg. 0342. The B16F10-Nex2 cell line was transduced with lentivirus vector pLKO.1 (B16-pLKO.1) as an empty control or in the pLKO.1-SOCS1i construction for SOCS1-silencing (B16shR-SOCS1) as previously described^16^. The human melanoma tissue sample, used as anti-HMB45 positive control by IHC, was obtained from the Hospital das Clínicas, University of São Paulo (HC-FMUSP). Cells were maintained in RPMI-1640 medium (Gibco, Minneapolis, MN; pH 7.2), supplemented with 10% heat-inactivated fetal calf serum, 10 mM Hepes (N-2-hydroxyethylpiperazine-N′-2- ethanesulfonic acid) and 24 mM NaHCO_3_ (all from GIBCO, Minneapolis, MN), and 40 mg/ml^−1^ gentamicin sulfate (Hipolabor, MG, Brazil).

### RNA microarray analysis

RNA was purified from 5 to 10^6^ exponentially growing B16F10-Nex2 and B16shR-SOCS1 cells using RNeasy total RNA kit (Qiagen, Hilden, Germany). RNA was used to synthesize the double stranded cDNA using reverse transcriptase and oligo-dT primer. The cDNA served as a template in an *in vitro* transcription (3′-IVT) robust reaction to yield amplified amounts of biotin-labeled complementary RNA (cRNA) or antisense mRNA, the microarray target. Fragments of cRNA are obtained using heat and Mg^+2^ and hybridized to *GeneChip*^®^ *Mouse Gene* 1.0 ST Array of Affymetrix according to protocols in the Expression Analysis Technical Manual (http://www.affymetrix.com/support/technical/manuals.affx).

### Transcriptome of differentially expressed genes in response to silencing of the SOCS1 gene

The transcriptome analysis was performed in duplicate using as control B16F10-Nex2 transfected with empty vector (B16-pLKO.1) and B16shR-SOCS1 silenced for SOCS1. The data were normalized with Robust Multi-array Average (RMA) algorithm available in the Affy R/Bioconductor software. Differentially expressed genes (DEGS) were identified by unpaired, significance analysis of microarrays (SAM) method *p* < 0.05, corrected by FDR (False Discovery Rate). Cluster analysis (clustering) was taken by HCL (Hierarchical Clustering) method with Euclidean distance measure and average linkage as a measure of distance between groups, available in MeV (MultiExperiment Viewer) program.

Functional analysis of identified DEGS used the IPA tool (Ingenuity Pathway Analysis, http://www.ingenuity.com). In addition to IPA, WebGestalt (WEB-based Gene Set Analysis Toolkit, http://bioinfo.vanderbilt.edu/webgestalt) program to classify DEGS was used. The parameters adopted in this analysis were: Organism: *Mus musculus*, Id Type: affy_mogene_1_0_st_v1; Statistics: Hypergeometric, significance Level: Top10, MTC: BH, Minimum: 2.

### Transduced tumor cell lysates

B16-pLKO.1 and B16shR-SOCS1 melanoma cells were harvested, and resuspended in PBS (5 × 10^6^ cells) with protease inhibitors. The cell suspensions were disrupted by 5-cycles of freezing–thawing using liquid nitrogen and 37 °C-water bath. Light microscopy and Trypan blue exclusion staining verified the method’s efficiency. Lysates were kept at −80 °C for later use.

### Western blotting analysis

Western blottings were run with proteins from total cell lysates (30 μg). The same lysates from B16F10-Nex2 and B16-shRSOCS1 cells were used in all Western blotting analysis. They were separated by 10% SDS-polyacrylamide gel electrophoresis and transferred to Immobilon P transfer membrane (Millipore, Darmstadt, Germany). The membranes were washed in Tris-buffered saline with Tween (10 mM Tris-HCl, pH 8, 150 mM NaCl, and 0.05% Tween 20) and blocked overnight at 4 °C with 5% nonfat milk in Tris-buffered saline with Tween 20. The blots were probed overnight at 4 °C with mAbs from Cell Signaling, Boston, MA; Bioss-bs336BR Woburn, MA; Santa Cruz, Dallas, TX; ABCAM, Cambridge, UK; as indicated. After 2 h incubation with horseradish peroxide-conjugated secondary antibody, immunoreactive proteins were detected by enhanced chemiluminescence (ECL; Amersham Biosciences, Little Chalfont, UK). Protein concentrations were determined by Bradford assay (Bio-Rad, Hercules, CA).

### PD-L1 on transduced tumor cells

B16-pLKO.1 or B16shR-SOCS1 tumor cells (10^6^ cells/well in 24-well plates) were collected, transferred to 1.5-mL microtube, washed and resuspended in PBS containing 10% BSA and incubated for 10 min on ice. After PBS washing they were incubated with PE-conjugated anti-murine PD-L1 antibody (BD Biosciences, San Jose, CA). After incubation on ice for 1 h in the dark, cells were washed and resuspended in 2% cold paraformaldehyde (wt/vol). Fluorescence was measured on FACSCanto flow cytometer (BD Biosciences, San Jose, CA) and data were analyzed by FlowJo (Tree Star Inc., San Jose, CA).

### Prophylactic Treatment and Tumor Development

Male C57Bl/6 (n = 10 per group), C57Bl/6-CD8^null^T and C57Bl/6-CD4^null^T (n = 3 per group), 6 to 8 weeks old, mice (CEDEME, UNIFESP) were housed under specific pathogen-free conditions. For prophylactic treatment, mice were immunized with 5 × 10^3^ B16-pLKO.1 or B16shR-SOCS1 viable cells subcutaneously into the left flanks (50 μL per mouse), 15 days before subcutaneous or intravenous challenge with B16F10-Nex2 melanoma cells. Subcutaneous challenges were made with 1 × 10^5^ tumor cells (95% viable by Trypan blue) in 0.05 ml of buffered saline into the right flanks (n = 10 per group). Tumor volume was calculated by: V = 0.52 × d^2^ × D (D, long diameter and d, short diameter). Animals were sacrificed as tumors reached a volume between 3,000 to 4,000 mm^3^. In the lung colonization (“metastatic”) model, mice were challenged with 5 × 10^5^ B16F10-Nex-2 cells i.v. (0.1 mL). Fifteen days later, mice had their lungs harvested, and the melanotic tumor nodules were counted with a loop. Histological evaluation of subcutaneous tumor tissue involved fixation in 10% neutral buffered formalin and staining with hematoxylin and eosin.

### Cytokine profile in splenocytes

Spleens were harvested from animals that have been immunized with B16-pLKO.1 or B16shR-SOCS1 viable cells as described above and were challenged s.c. and i.v. with B16F10-Nex2 cells. Fifteen days after the challenge, splenocytes were cultured for 3 days in the presence of B16F10-Nex2 tumor cell lysate. Supernatants were collected and subjected to cytokine analysis by flow cytometry using the FACSCANTO II (CBA-BD Biosciences, San Jose, CA) and FlowJo software (Tree Star Inc. Ashland, OR).

### Histological analysis

Mice challenged s.c. with B16F10-Nex2 cells were sacrificed as tumors reached 500 mm^3^ (7–10 day). Mice immunized s.c. with B16shR-SOCS1 (no tumor growth) were sacrificed 10 days afterwards. The tissue at the site of tumor cell grafting was excised, fixed in formalin, dehydrated through graded alcohol and xylene and embedded in paraffin; sections were cut and stained with hematoxylin/eosin.

### T-CD8 and NK1.1 cells in the tumor infiltrate

Mice grafted s.c. with B16F10-Nex2 and with B16shR-SOCS1 cells were sacrificed 15 days after challenge and the tissue at the site of tumor cell grafting was excised, filtered through a 40 μm diameter strainer (BD Bioscience, Franklin Lakes, NJ) and washed with PBS. After washing, cells were incubated with PE-conjugated anti-murine NK1.1 antibody and FITC-conjugated anti-murine T-CD8 antibody (BD Biosciences, San Jose, CA). After incubation on ice for 1 h in the dark, cells were washed and resuspended in 2% cold paraformaldehyde (wt/vol). Fluorescence was measured on FACSCanto flow cytometer (BD Biosciences, San Jose, CA) and data were analyzed by FlowJo (Tree Star Inc., San Jose, CA).

### Immunohistochemistry

Spleens and lungs were harvested from animals that have been immunized with B16-pLKO.1 or B16shR-SOCS1 viable cells as described above and were rechallenged i.v. with B16F10-Nex2 cells. Immunohistochemistry was performed using a microwave-based antigen retrieval technique. Previously, the tissue was deparaffinized, rehydrated and submitted to citrate buffer, pH 6.0, plus the antigen retrieval solution at 98 °C. The endogenous peroxidase activity was blocked with 3% hydrogen peroxide, and Protein Block Solution (DAKO, Glostrup, Denmark). The slides were incubated overnight at 4 °C with specific primary antibodies (1/100), anti-HMB-45, anti-CD11b, anti-CD4, anti-CD8, anti-PD-L1 or anti-CD3 (Abcam, Cambridge, MA), anti-FOXP3 (Santa Cruz, Dallas, TX), followed by incubation with the labeled polymer (Dual Link System-HRP; DAKO, Glostrup, Denmark) using two sequential 30-min incubations at room temperature. Brown staining was obtained with 1- to 3-min 3,3′-Diaminobenzidine (DAB) incubation with EnVisionTM FLEX Substrate Working Solution (DAKO, Glostrup, Denmark).

The slides were counterstained with 5% hematoxylin, and then examined by light microscopy. For each section, 10 microscopic fields (corresponding to a total area of 1.6 mm^2^) were examined. The area marked was measured with an image processing software (Image Pro Plus^®^ version 7.01, Media Cybernetics, Warrendale, PA) and the optical density was calculated by the following formula: OD = log (max intensity/Mean intensity), where max intensity = 255 for 8-bit images.

### Statistical analysis

Each experimental group consisted of 3, 5 or 10 animals. Values were analyzed for statistical significance using Prism (GraphPad, San Diego, CA). Other statistical tests used were Two-way ANOVA, Student t test and Kaplan-Meyer’s and Log-rank tests. In all studies, p < 0.05 was considered statistically significant.

## Additional Information

**How to cite this article**: Berzaghi, R. *et al*. SOCS1 favors the epithelial-mesenchymal transition in melanoma, promotes tumor progression and prevents antitumor immunity by PD-L1 expression. *Sci. Rep.* **7**, 40585; doi: 10.1038/srep40585 (2017).

**Publisher's note:** Springer Nature remains neutral with regard to jurisdictional claims in published maps and institutional affiliations.

## Supplementary Material

Supplementary Dataset 1

Supplementary 1
