# Erratum: SOCS1 favors the epithelial-mesenchymal transition in melanoma, promotes tumor progression and prevents antitumor immunity by PD-L1 expression

**DOI:** 10.1038/srep42827

**Published:** 2017-02-21

**Authors:** R. Berzaghi, V. S. C. Maia, F. V. Pereira, F. M. Melo, M. S. Guedes, C. S. T. Origassa, J. B. Scutti, A. L. Matsuo, N. O. S. Câmara, E. G. Rodrigues, L. R. Travassos

Scientific Reports 7: Article number: 4058510.1038/srep40585; published online: 01 12 2017; updated: 02 21 2017

Affiliation 1 was incorrectly listed as ‘Experimental Oncology Unit, Department of Microbiology, Immunology and Parasitology, University of São Paulo, São Paulo, Brazil’ in the original version of the Article. The correct affiliation is listed below:

Experimental Oncology Unit, Department of Microbiology, Immunology and Parasitology, Federal University of São Paulo, São Paulo, Brazil.

In Addition, E.G. Rodrigues was incorrectly listed as being affiliated with ‘Laboratory of Cancer Immunobiology, University of São Paulo, São Paulo, Brazil’.

The correct affiliation is listed below:

Laboratory of Cancer Immunobiology, Department of Microbiology, Immunology and Parasitology, Federal University of São Paulo, São Paulo, Brazil.

These errors have now been corrected in the HTML and PDF versions of this Article.

