# Supplementary material for: SOCS1 favors the epithelial-mesenchymal transition in melanoma, promotes tumor progression and prevents antitumor immunity by PD-L1 expression
Source: Sci Rep. 2017 Jan 12;7:40585. doi: 10.1038/srep40585 (PMC5227698; doi:10.1038/srep40585)
Supplement: Supplementary 1 [file srep40585-s2.doc]

**Supplementary 1**

**SOCS1 favors the epithelial-mesenchymal transition in melanoma, promotes tumor progression and prevents antitumor immunity by PD-L1 expression.**

Berzaghi R^1^, Maia VSC^5^, Pereira FV^2^, Melo FM^3^, Guedes MS^1^, Origassa CST^2^, Scutti

JB^7^, Matsuo AL^6^, Câmara, NOS^4^, Rodrigues EG^2^ and Travassos LR^1, 5*^

Legend

**Supplementary 1.** The Whole set of differentially expressed genes between B16F10-Nex2 cells and B16shR-SOCS1 cells. Red, upregulated DEGs; Green, downregulated DEGs.
